# A Systematic Review of Educational Interventions and Their Impact on Empathy and Compassion of Undergraduate Medical Students

**DOI:** 10.3389/fmed.2021.758377

**Published:** 2021-11-08

**Authors:** Prianna Menezes, Salman Y. Guraya, Shaista Salman Guraya

**Affiliations:** ^1^Royal College of Surgeons Ireland, Bahrain RCSI–Medical University of Bahrain (MUB), Busaiteen, Bahrain; ^2^University of Sharjah, Sharjah, United Arab Emirates

**Keywords:** compassion, empathy, medical students, communication skills, mindfulness, technology-enhanced learning

## Abstract

**Introduction:** A compassionate and patient-centered care leads to improved clinical outcomes. Promoting empathy and compassion of medical students is a forerunner of their well-being, emotional stability, and a patient-centered care. However, there is slender evidence about best educational interventions that can inculcate empathy and compassion skills. Our objective was to conduct a systematic review of research evaluating the associations between spectrum, effectiveness, frequency of teaching modalities and their outcomes on compassion and empathy to highlight best practices.

**Methods:** We searched the Web of Science, PubMed, Scopus, and EBSCO Host on 22nd July 2020. We adapted our search strategy from a previously published systematic review on education for compassion and empathy. Selected studies were required to have used unique educational interventions for promoting empathy and compassion of medical students. The research questions were based on Participants (medical students), Intervention (empathy and/or compassion related teaching), Comparison, and Outcome.

**Results:** We analyzed 24 articles from the initial yield of 2,861. Twenty-two were quantitative studies with a mean of 12.8 on MERSQI. Twelve were randomized controlled trials while 5 measured outcomes with single group pre- and post-tests. There was no association found between duration, frequency and complexity of an educational intervention and its effectiveness. Twenty used multimodality curricula, and of those 18 reported statistically significant positive improvement in empathy, while 3 of 4 single modality were effective. Only three studies looked for long-term effects of educational interventions. Fourteen studies evaluated Kirkpatrick's level one (self-reported knowledge), 2 level three (behavior), and 6 level four (patient outcomes). We identified six major educational constructs of teaching empathy and compassion; communication, mindfulness, early clinical exposure, technology-enhanced learning, comics and arts and culture.

**Discussion:** Our review couldn't identify a standard teaching construct in place and highlighted that different teaching tools carry similar impact in promoting compassion and empathy and a sustainable program rather than a single training activity is essential.

## Introduction

The quality of the interaction between physicians and patients influences patient outcomes in clinical settings (1, 2). A fundamental pillar of the healthcare mission is based upon compassionate care that forecasts greater patient trust and satisfaction, superior patient-physician connections, and better patient outcomes (2, 3). Within healthcare organizations, compassionate care entails diverse responsibilities of healthcare professionals in explaining their roles, establishing rapport, and in spending time on attentively listening to the patients' concerns (4). Compassionate healthcare—a key competency is characterized by reflections of others' concerns, respect for persons, and contextualized understanding of the patient as a key player of healthcare systems (5).

The two leading elements of patient centered care include empathy and compassion. Empathy refers to “a capacity to understand but without joining the feeling of the patient” (6) while compassion refers to “the feeling that arises in witnessing another's suffering and that motivates a subsequent desire to help” (7). In the medical field, empathy pertains to a multifaceted strand with moral, cognitive and behavioral enlightenment (8). When applied with objective reasoning, empathy promotes the impact of medical care and facilitates physician-patient communication (9). Empathy, being a multi-construct concept, includes, but not limited to, physician-patient interactions, interprofessional practice, self-compassion, empathy for others' pain, professional identity formation, stress awareness, and self-reflection and communication. Empathetic communication in patient-physicians interactions fosters information exchange and the impact of understanding and adherence to management plans, which lead to an early return to work, pain relief, mood elevation, and improved functional status of patients (10). Both empathy and compassion are generally considered to be interchangeable terms. However, empathy (understanding of patient feelings) is necessary to trigger compassion (emotional response including actions to alleviate patient sufferings) (11).

There is a compelling evidence in literature that treating patients without compassion can lead to deleterious outcomes (12). An absence of compassionate care results in poor quality of care and higher risk of complications through medical errors (13). Unfortunately, despite an explicit emphasis on the vital role of compassionate patient care, healthcare professionals often miss opportunities to be compassionate, rather they pay attention to biomedical data and management plans. From another perspective, sustaining compassion in medicine is hard as “compassion fatigue” prevails in ~20–70% of healthcare professionals (14). Compassion fatigue refers to loss of compassion in healthcare providers due to work related stress (15). Compassion fatigue certainly impairs the ability of healthcare providers in providing compassionate care which is a fundamental pillar inpatient-centered service (16). Educators have argued that a great majority of medical students enter health care with a strong set of espoused ideals for providing high-quality, patient-centered care (17). Unfortunately, during their training, medical students witness dissonance between the personal and professional conducts of their faculty and clinical teachers, students become more frustrated, less empathetic and more distanced from patients (18).

By and large, the ethos of most health care professional curricula uses typical biomedical models that primarily focus on teaching, training, and practice of clinical medicine with less emphasis on patients' psycho-social well-being (19). In the absence of a rigorous integration of biomedical knowledge with the understanding of human behaviors will potentially further detach medical and health sciences students and healthcare professionals from the patient's emotions and contextualized perspectives (20). Likewise, empathy plays a vital role in interprofessional practice where physicians from various disciplines work together toward the safe and effective patient-related clinical outcomes by shared decision-making and by regular consultations among healthcare teams and patients (21). This interprofessional practice alleviates patients' anxiety, stress, and uncertainty. From another perspective, self-awareness and self-regulation with empathy facilitates patient-doctor therapeutic interactions by evolving physicians' self-discovery (22). Among the practicing physicians, the phenomenon of self-discovery is an ingredient to professional identity formation (23). An empathetic professional identity formation is considered to be an effective tool for therapeutic actions in the healthcare system (24). In summary, empathy embodies a constellation of inter-related and interchangeable attributes that collectively lead to improved positive patient-related outcomes and the quality of care.

In clinical practice, a compassionate care has shown a strong association between improved clinical outcomes, quality of life and well-being (25). Among health professionals, empathetic, and compassionate care helps in mitigating the risk of burnouts (26). From the neuroscience perspective, a study on the functional magnetic resonance imaging has shown that empathy activates distinct pain centers of the brain; whereas focusing on compassion activates the reward pathways (27). These findings propound that a synchronous teaching and practice of empathy and compassion enhance patient and clinician well-being. Such outcomes may be accomplished with great success if educators can inculcate structured training programs for empathy and compassion in undergraduate and residency programs. However, there is a compelling evidence of empathy decline during the course of educational and training programs (28) and there is an urgent need for developing evidence-based curricula that can secure a sustained change in attitudes and behaviors (29).

A wealth of teaching pedagogies has been used to develop empathy, compassion and respect for patients in medical students (30–33) These include, but not limited to, patients' narratives and creative arts, drama workshops, communication skills, reflective writing, video-based learning and experiential learning (34–37). Lastly, the impact of role modeling and hidden curriculum in teaching compassion and empathy is well-established (38–40). There is little evidence about how the learning contexts, including the structure and delivery of medical curricula, influence the understanding of medical students about empathy and compassion (41). However, some patient-reported studies about compassion and empathy have shown the influence of educational interventions on medical students in enhancing their approach toward patients and their families (42).

The analysis of existing body of literature emphasizes a need to introduce a standard teaching modalities within medical curricula that can enrich traits of empathy and compassion in undergraduate medical students. Currently, there is scarce evidence of horizontal or vertical integration of teaching programs of empathy and compassion in undergraduate medical curricula (43, 44). Furthermore, there is little evidence about the effectiveness and quality of teaching programs for empathy and compassion. We conducted this systematic review of the literature to summarize and report the published work on educational interventions for empathy and/or compassion curricula in undergraduate medical students. This review also aims to highlight best practices to implement an evidence-based empathy and/or compassion curriculum in undergraduate medical training.

## Materials and Methods

In our study, we used the Preferred Reporting Items for Systematic Reviews and Meta-Analyses (PRISMA) guidelines (45). The PRISMA tool provides an evidence-based minimum set of data for a standard reporting in systematic reviews and meta-analyses.

### Research Objectives

Our research questions were based on Population, Intervention, Comparison, and Outcome (PICO) (46) as shown in Box 1.

Box 1 **The checklist used for screening abstracts to determine the eligibility of studies for their full-text analysis.**

**Population**

*Does this study look at medical students? YES/NO

*If NO exclude*

**Intervention**

Does this study use compassion/empathy/caring training? YES/NO

*If NO exclude*

Does this study train medical students to compassionate or empathetic strategies? YES/NO

*If NO exclude*

Does this study include information regarding the content of the compassion or empathy or caring training? YES/NO

*If NO exclude*

**Comparison**

Present? YES/NO

Absent? YES/NO

**Outcome**

Does this study include outcome measures related to the compassion/empathy/caring training? YES/NO

*If NO exclude*

Does this study isolate the outcomes for medical students? YES/NO

*If NO exclude*

We conducted this systematic review with two major objectives.

1. To describe and summarize the published literature about empathy and/or compassion curriculum in undergraduate medical education.
2. To summarize and highlight the best practices to implement an evidence-based empathy and/or compassion curriculum in undergraduate medical education.

### Literature Search Strategy

We searched four major electronic databases of Web of Science, PubMed, Scopus and EBSCO Host for the English-language articles, published during 2015–2020. Our search strategy was adapted from a previously published systematic review on education for compassion and empathy (47, 48). We tweaked this work further to explicitly focus on medical students. The final search was performed on July 22nd, 2020 and a detailed search strategy is attached as Appendix 1. We looked into three core concepts and their associated Medical Subject Headings (MeSH) terms and keywords: Compassion and empathy, medical students, and educational interventions (Compassion OR Empathy OR Caring AND Medical students AND Education OR Training OR Workshop OR Simulation). A hand search of reference lists of the relevant articles yielded some more studies which were included in the final list of selected articles.

### Data Collection, Eligibility Criteria, and Selection of Articles

We included original research studies that (i) carried out research on undergraduate medical students; and (ii) showed a clear educational intervention for empathy and/or compassion; and (iii) measured educational outcomes about compassion and/or empathy. These articles showed educational outcomes after training interventions to improve empathy (the understanding component) and compassion (i.e., the action component). The original studies included controlled trials, randomized controlled trials, pre- /post-test and post-test only designs. Review and editorial articles, commentaries, experts' opinion, short communications, and letter to the editor were excluded from our search. PM and SSG reviewed the titles and abstract independently and separately using PICO criteria. Any discrepancies were resolved by SG.

### Data Extraction and Data Synthesis

Two researchers (PM and SSG) thoroughly scanned the full text of articles that met inclusion criteria and then using a standard data extraction form charted the required information. The researcher SG independently reviewed the entire process and filled gaps in data mining, data extraction and synthesis. We considered the following components during data extraction; types and designs of studies, primary objectives of studies, quality of studies, invitees' study level, response rate, self-reported or objective measurements in each study, and type, duration, frequency, skills taught and modality of educational interventions. We also recorded the outcomes according to the following four levels of the Kirkpatrick's model (49);

1. Self-reported changes in knowledge, skills, and attitude
2. Changes in knowledge
3. Changes in behavior
4. Patient-reported outcomes

We identified and classified the primary outcomes of the selected studies according to Kirkpatrick's level. A number of studies assessed multiple competencies such as empathy and compassion and self-compassion, and the highest-level outcome was considered as the primary outcome. While, in the studies where the primary outcomes did not relate to compassion or empathy, we used the highest-level empathy-specific variable as the primary outcome (Boxes 1, 2).

Box 2 **The data mining rubric used in our study to record characteristics of each study (*n* = 24).**

**First Author**

**Title**

***Year*** **of Publication**

**Country**

**Study Design**

- Pre-post curriculum evaluation
- Randomized controlled trial
- Controlled trial
- Other

**Population**

- Number of students
- Year of students

**Curriculum** Hours

Number of sessions

**Pedagogical approach**

- Didactics
- Small group discussions
- Written/verbal reflections
- Simulation
- Standardized patient practice
- Apprenticeship/mentoring/service learning
- Video/Demonstration
- Other (Virtual hangouts, blogs, hot spotting etc.)

**Compassion/empathy related Outcomes**

- Self-reported changes in knowledge, skills, and attitudes
- Knowledge
- Behaviors
- Patient outcomes

### Effectiveness of Educational Interventions

To identify best educational practices for teaching compassion and/or empathy, we determined the effectiveness of interventions in the selected studies, where effective was defined as a statistically significant improvement in primary educational outcomes as measured by *p*-value or effect size, where applicable. A *p* < 0.05 and an effect size >0.25 was considered significant. This rule was applicable only for quantitative studies with a control group design or a single group pre and post-test design.

### Quality Assessment

We used Medical Education Research Study Quality Instrument (MERSQI), a tool designed for evaluation of quantitative educational research studies (50). The MERSQI checklist has 10 items in 6 domains: study design, sampling, type of data, validity evidence, data analysis, and type of outcomes with a maximum score of 3 in each domain. A study can have a maximum MERSQI score of 18 (highest quality). PM and SSG individually scored each study and in case of score discrepancies, SG assessed the scoring and discussed and made the final decision.

### Quality Assurance

All researchers (PM, SSG and SG) objectively reviewed the workflow of selection of studies. In case of discrepancies, the researchers reached consensus by comparing the studies with inclusion criteria and key words. The discrepancies, inconsistencies and controversies were resolved with consensus until all the concerns were resolved.

## Results

**Figure 1:**
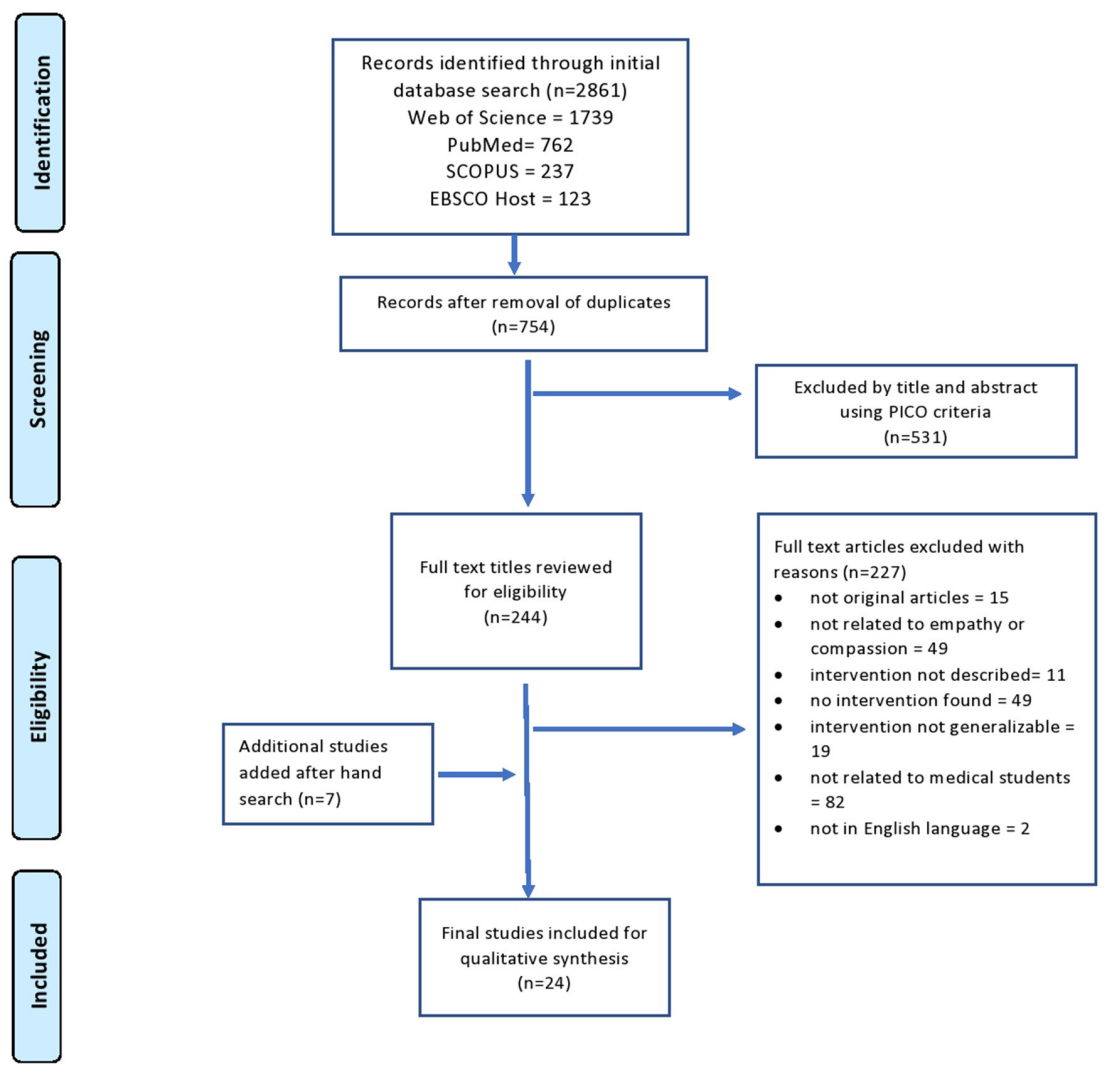
Flow diagram of the preferred reporting items for systematic reviews and meta-analysis (PRISMA) guidelines for the selection of articles in this study.

Figure 1 outlines the workflow and algorithm of studies selection in this study. Our initial search yielded 2,861 studies. After removing duplicates, we screened 754 titles and abstracts. This led to the identification of 244 articles using PICO for a detailed full-text review. This helped us to exclude another 227 articles whose content did not meet the inclusion criteria. We reviewed reference lists of the included articles to identify additional studies for potential inclusion, used electronic citation tracking, and consulted the librarian. This hand search yielded 15 review articles with 7 additional articles for inclusion. Finally, authors agreed on a list of 24 articles (23, 51–73), that had used 24 unique educational interventions either *de novo*, validated or adapted from previous publications and explicitly met inclusion criteria of our research. The summarized and comprehensive information about each article is presented in Table 1.

**Table 1:** A tabulated summary of the 24 studies in this systematic review.

**Study**	**Study population**	**Study design**	**Curriculum design**	**Empathy topics addressed**	**Primary outcome (effect size and *P*-value where available)^**a**^**	**Quality assessed (MERSQI)^**b**^**
**Physician-patient interaction**						
Beard et al. (51)	***N*****:** 10 **Level of training:** Third year	Controlled Trail 2 groups	**Modality:** Longitudinal integrated clerkships—VALUE **Frequency/Duration:** 10 months	A respect for a patient's values and preferences/a clear patient physician communication/A well-coordinated care	**Patient outcome**–A greater sense of satisfaction reported by VALUE patients with their health care providers in terms of explanations provided, knowledge of patients' history, and their best interests (*P* < 0.05)	16.5
Collins et al. (52)	***N*****:** 45 **Level of training**: Third and fourth year	Controlled trial (control-no intervention)	**Modality**: Student hot spotting/IPE/apprenticeship/supervision **Frequency/Duration**:6 months	Patient centered approach/Partnership for a personalized self-management plan	**Knowledge:** ATHI, JSE; A higher post-test score in terms of self-efficacy and empathy (participants Vs. controls) (*P* = 0.05).	10.5
D'souza et al. (53)	***N*****:** 82 **Level of training:** Second year	RCT	**Modality**: Didactic PowerPoint, video clips, and roleplay and simulation **Frequency/Duration**: Single session −2 h	Empathetic communication	**Self-report**: JSE: a difference in empathy score (control vs. intervention) (*p* = 0.014) with a decline at 3-week follow-up (*p* = 0.020)	12
Kataoka (62)	***N*****:** 69 **Level of training**: Year 1–6	Single group, pre and posttest	**Modality:** didactics case-based discussions; simulation with standardized patients, feedback provisions **Frequency/Duration:** Three 4-h workshops over a period of two years	Communication skills and medical interviewing	**Self-report:** JSE: an immediate significant increase (SD = 10.0) in post-test mean score (*p* < 0.0001), however, the mean score bounced back to the pre-test level in year 5 (SD =1 2.9) and year 6 (SD = 13.8)	10
Modi et al. (65)	***N*****:** 188 **Level of training**: First to third year	Controlled trial (control-no intervention)	**Modality:** Service learning experience—student run free clinic—socialization-mentorship **Frequency/Duration**: Weekly student run clinics over a period of 3 years	Early and consistent exposure to poor and underserved Patients—hidden curriculum—implicit to explicit	**Self-report**: JSE: A drop in mean empathy scores for both volunteers (2.2 points) (*P* = 0.07, effect size = 0.20), and non-volunteers (3.1 points) (*P* = 0.009, effect size > 0.25)	10
Smith et al. (69)	***N*****:** 122 **Level of training**: First year—third year	Single group pre- and post-test	**Modality**: Online surveys and computerized tasks **Frequency/Duration**: At start and end of each academic year for first 3 years of medical school	Pain visual analog scales, being sensitive to others' pain and how to understand others' emotions (video ratings of individuals expressions of pain and RMET)	**Behavior**-RMET; An significant improvement in accuracy in recognizing others' emotional states, and a decrease in reaction time in longitudinal measurements (*p* < 0.001) **Self-report**-JSE score decreased over training (*p* < 0.01) while QCAE revealed an improvement in different empathy components; cognitive (perspective taking,) and affective (emotion contagion) (*p* < 0.05)	15.5
Wundrich et al. (72)	***N*****:** 158 **Level of training**: Third year	RCT	**Modality**: Videos, simulation, and OSCE **Frequency/Duration**: 2 sessions (2.25 h each)	Physician–patient relationship, empathy skills, and behaviors	**Patient outcome**-by standardized patients and experts, significantly higher empathy score and ratings as compared to control group (*p* < 0.05) **Self-report**-JSE: no significant difference (*p* = 0.13)	16
Ruiz-Moral et al. (66)	***N*****:** 115 **Level of training**: Third year	Single group, pre- and post-test	**Modality:** Multiple didactic, reflective, and interactive workshops and simulated patients encounters **Frequency/Duration**: 6 weeks course	Contextual and emotional clues/empathetic response tailoring/communication process to identify the feeling produced by the empathetic responses	**Patient outcome**-Progressive improvements over longitudinal period of time spanning all the domain and skills of communications by both OE (32.4%) and SP (38.3%) (*p* < 0.001)	13.5
Singh et al. (68)	***N*****:** 93 **Level of training**: Second year	RCT 2 tests (CDG and VSG) and 1 control	**Modality:** Low-fidelity simulation techniques (case discussions and a video show), interactive lectures, video show, and demonstration were used **Frequency/Duration**: 4 sessions in a week (4.5 h)	Emotional, social, and financial consequences of HAI on patients and their families	**Knowledge**-significant change in knowledge test score (*p* = 0.016) among the groups **Self-report**-TEQ: significant difference in post-test empathy scores among the groups (*p* = 0.026) CDG (*p* = 0.011), VSG (*p* = 0.046) had significantly better empathy scores vs. control group	12.5
Foster et al. (60)	***N*****:** 70 **Level of training**: First year	RCT 2 tests and 1 control	**Modality:** Online text-based interface for virtual patient (VP) interaction **Frequency/Duration**: Single session	Empathetic Communication and Feedback VP: Depression portrayed by control VP A VP with a simulation backstory of patient shadowing, or An immediate empathy-feedback VP	**Patient outcome**-StP ratings; A significantly higher scores on empathy-feedback and backstory VP groups vs. control VP group (*P* < 0.0001) Trained assessors: A promising response of students in eliciting empathetic opportunities in empathy-feedback VP group vs. backstory VP and control VP groups (*P* = 0.0005)	17
LoSasso et al. (63)	***N*****:** 70 **Level of training:** Third year	RCT	**Modality:** Small group discussion of EMR use, the SALTED technique (set-up, ask, listen, type, exceptions, documentation), and role-plays **Frequency/Duration**: 1 h	Training in EMR Specific Communication: empathetic engagement while history-taking and doctor patient interaction	**Patient outcome**-Significantly higher mean SP ratings for intervention vs. control group (*P =* 0.05) **Self-report**-JSE: non-significant change in mean empathy score for intervention (*P* = 0.57) vs. control group (*P* = 0.41)	
Yu et al. (73)	***N*****:** 82 **Level of training**: First and second year	RCT	**Modality**: Didactic **Frequency/Duration**: 1 h class	Interpreting micro and subtle facial expressions	**Self-report**-significant post intervention increase of mean METT (29.3%) and SETT (36.2%) scores (*P* < 0.001)	12
Demiroren et al. (56)	***N*****:** 190 **Level of training**: Fourth and fifth year	RCT	**Modality:** Small group case-based discussions, guided written, and verbal reflections **Frequency/Duration**: 2.5 weeks course	Appropriate professional values and behavior; Patient-physician interaction	**Self-report**-BMI; JSE; PSCOM-PQ; BPTI*;* a statistically significant impact of training on conscientiousness (*p* = 0.003), openness to experience (*p* = 0.033), compassionate care (*p* = 0.018) and standing in the patient's shoes (*p* = 0.036); while students reported verbal reflections more beneficial vs. written	13.5
Tsao and Catherine (70)	***N*****:** 25 **Level of training**: First and second year	Single group post-test only (qualitative)	**Modality**: Didactics, online study material, comic video recordings, in class guided written reflections and focus group discussions **Frequency/Duration**: 4-h single session	Struggles of diabetes patient; how to bring behavioral change, reduce burnout, address fear of insulin initiation, avoid guilt, curb denial, and frustration with complications	**Self-report**-Assessment of personal reflections revealed more empathy, better able to reflect and make meaning from work JSE: mean JSPE scores baseline (116.4) and after watching comic videos, reflections (117.2) and focus group discussions (119.6)	NA
**Interprofessional practice and professional identity formation**						
Davison (55)	***N*****:** 170 **Level of training**: First year	Single group, post-test only	**Modality:** Student supervision by an HCA mentor in an early clinical exposure curriculum **Frequency/Duration**: 3 days	Incorporate human values while underpinning Interprofessional practice (IPP)	**Self-report:** Reflections narrated more able to empathize, better equipped and confident and appreciate IPP	11
Chrisman-Khawam (23)	***N*****:** 64 **Level of training**: Undergrad students	Single group, post-test only (qualitative)	**Modality:** Service-learning experience—student run free clinic—socialization-mentorship–reflective practice **Frequency/Duration**: Weekly winter season clinics	Model of patient-physician relationships/interprofessional relationships/professional identity formation	**Self-report**: More introspective and a sense of connection to patients on a human level	NA
Schweller et al. (67)	***N:*** 166 **Level of training:** First year	Single group, pre and post-test	**Modality:** Patients and physicians' interviews, role modeling, supervised hospital visits, analysis of videotaped simulated consultations **Frequency/Duration:** Weekly session over 4 months	Health and Medicine (H&M): professional identity formation by incorporating desired Values and virtues	**Self-report**-JSE: Improved mean empathy scores (117.9 vs. 121.3) (*p* < 0.001)	9.5
**Mindfulness and self-compassion**						
Fernando et al. (59)	***N*****:** 83 **Level of training**: Third year	RCT	**Modality**: Didactics–mindfulness based exercises—simulation—role play **Frequency/Duration:** 2 h single session	Emotions and clinical decision making/Self-compassion/Mindfulness/speech on civic service	**Behaviors; Objective**: A varying fluctuations of time allocated to each patient by participants with lower self-compassion vs. a consistent time allocation to each patient by persons with high self-compassion **Self-report:** SCS, MCSF-C, TMS, B-DES, VAS–Mindfulness led to an increased patient liking and caring in persons with lower self-compassion vs. a greater helping behavior in persons with a higher self-compassion. A promising enhancement of compassionate responses in medical students after a brief mindfulness induction (*P* < 0.05)	15
du Vaure et al. (57)	***N*****:** 299 **Level of training**: Fourth year	RCT	**Modality**: Simulation of interpersonal problems–Balint group **Frequency/Duration**: 7 weekly sessions over a period of 2 months	Solution to interpersonal problems during physician patient interaction	**Patient outcome:** CARE: Non-significant difference in mean CARE score (Intervention vs. control groups) (*P* = 0.49) **Self-rate**-JSE: an increase in score for intervention vs. a decrease in score for control from baseline to follow-up [*P* = 0.031]	16.5
van Dijk et al. (71)	***N*****:** 167 **Level of training:** First year of clinical clerkships (second/third year)	RCT	**Modality:** Didactic class room teaching **Frequency/Duration**: Eight weekly 2-h sessions	**MBSR**: Stress awareness and mindfulness: communication, work life balance, and recognizing boundaries	**Self-report**-BSI, MHC-SF, LiSat-9, JSE, FFMQ, IBI, MBSR group—a small reduction of psychological distress (*P =* 0.03) and dysfunctional cognitions (*P =* 0.05), while a moderate increase of positive mental health (*P* = 0.002), life satisfaction (*P* = 0.01), and mindfulness skills (*P =* 0.05) vs. CAU over a 20-month follow up. No significant change on empathy (*P =* 0.18)	13
Mascaro (64)	***N*****:** 32 **Level of training**: Second year	RCT (control-wait list)	**Modality:** Didactic CBCT course, guided audio recordings, at home meditation practice **Frequency/Duration**: 1.5 h once per week for 10 weeks	Compassion meditation protocol—stability of mind, insight to inner world of thoughts and feelings, self-compassion, equanimity, appreciation and gratitude cultivation, empathy, and compassion for others	**Self-report**-CLHS; R-UCLA, DASS; PSS; SUI–increased compassion, decreased loneliness, and depression (paired *t*-tests, *p* > 0.05)	11.5
Danilewitz et al. (54)	***N*****:** 30 **Level of training**: First and second year	RCT (Control-wait list WL)	**Modality**: Medical student led peer program, homeworks **Frequency/Duration**: 8, 1.15 h weekly sessions	Adapted MBSR program: Stress awareness and mindfulness: communication, work life balance, and recognizing boundaries	**Self-report**-DASS; JSE; FFMQ; SCS; AAS–MMP; a significant pre-/post-test reductions in levels of stress (*p* = 0.019), increase in self-compassion (*p* = 0.024) and altruism (*p* = 0.033) and changes in two facets of mindfulness: describe (*p* = 0.05) and non-react (*p* = 0.034). Significantly higher MMP vs. WL post-test scores on FFMQ (*p* = 0.026)	11.5
Duke et al. (58)	***N*****:** 259 **Level of training**: Third year	Single group, pre- and post-test	**Modality:** Virtual hangouts–tutorials, small group discussion, reflections, blogs on VCR, LM, and SM **Frequency/Duration**: Virtual meetings every 8–12 weeks over 1 year	Appropriate professional values and behavior; empathy, and self-reflection	**Self-report**-JSE no change in pre-/post-test mean score, while a statistically significant increase in GRAS scores (*p* < 0.001) Assessment of blogs highlighted that sharing of personal narratives foster reflective ability and reflective practice	10.5
**Arts & humanities**						
Graham et al. (61)	***N*****:** 68 **Level of training:** NR	Controlled trial (Control- no intervention)	**Modality**: Didactic seminars, in class discussions, about films and art **Frequency/Duration:** 10 in class contact hours in an elective course	Humanities course: sociocultural studies, history of western medicine, and visual arts and literature	**Self-report**-JSE Favorable empathy scores after humanities course (*P* = 0.03)	10

*VALUE, Veterans Affairs Longitudinal Undergraduate Medical Education; HCA, Health Care Assistant; JSE, Jefferson Scale of Empathy; SCS, Self-Compassion Scale; MCSF-C, Marlowe- Crowne Short Form C; TMS, Toronto Mindfulness Scale; B-DES, Brief Differential Emotions Scale; VAS, Visual Analog Scale; CARE, Consultation And Relational Empathy Measure; BSI, Brief Symptom Inventory; MHC-SF, Mental Health Continuum-Short Form; LiSat-9, Life Satisfaction Questionnaire; FFMQ, Five Facet Mindfulness Questionnaire; IBI, Irrational Beliefs Inventory; MBSR, Mindfulness Based Stress Reduction Training; CAU, Clerkships As Usual; QCAE, Questionnaire of Cognitive and Affective Empathy; OE, External observer; SP, Simulated patients; HAI, Healthcare Associated Infections; CDG, Case Discussion Group; VSG, Video Show Group; EMR, Electronic Medical Records; METT, Micro Expression Training Tool; SETT, Subtle Expression Training Tool; CBCT, Cognitively-Based Compassion Training; CLHS, Compassionate Love for Humanity Scale; R-UCLA, UCLA Loneliness Scale; DASS, Depression Anxiety and Stress Scale; PSS, Pittsburgh Sleep Scale; SUI, Substance Use Inventory; MMP, Mindfulness Meditation Program; AAS, Adapted Altruism Scale; BMI, Beliefs toward Mental Illness Scale; PSCOM-PQ, Penn State College of Medicine Professionalism Questionnaire Student Form; BPTI, Basic Personality Traits Inventory; GRAS, Groningen Reflection Ability Scale; VCR, Virtual Classrooms; LMS, Learning Management system; SM, Social Media*.

a *A bold outcome indicates that a statistically significant positive effect on the primary outcome was reported*.

b *The MERSQI is scored out of a possible 18, with higher scores indicating higher-quality studies*.

A maximum number of 9/24 (37.5%) articles were published in 2017, while 11/24 (45.8 %) studies originated from the USA. The sample size varied greatly across the selected studies, ranging from 10 to 299 participants. Our research included a total of 2,657 respondents with a mean of 110. Table 2 outlines the range of study designs that were used in the selected 24 studies. Most studies (21/24; 87.5%) were single-center, 11 (50%) were randomized controlled trials (54, 56, 57, 59, 60, 63, 64, 68, 71, 72), 4 (16.6%) controlled trials. (51, 52, 61, 65) Five (20.8%) studies measured outcomes with single group pre- and post-tests. (58, 62, 66, 67, 69) Our research identified 22/24 studies with their reported quantitative data that allowed us to calculate their MERSQI scores as shown in Table 3. All studies used statistical means to evaluate effectiveness. Collectively all studies have a total MERSQI score of 283 with a mean of 12.8. The study by Foster et al. (60) secured the highest MERSQI score of 17 from a maximum of 18. Finally, 10/22 (45.5%) studies scored ≥13 on MERSQI checklist.

**Table 2:** The range of study designs used in the selected studies about empathy and compassion in medical students (*n* = 24).

**Study design**	**Number (percentage)**	**Publications references**
Single group, post-test only	3 (12.5%)	(23, 55, 70)
Single group, pre-, and post-test	5 (20.8%)	(58, 62, 66, 67, 69)
Controlled trials	4 (16.6%)	(51, 52, 61, 65)
Randomized controlled trials	12 (50%)	(8, 10, 11, 13, 14, 17, 18, 22, 25, 26)
Two or more institutions	3 (12.5%)	(56, 57, 69)

**Table 3:** The quality of the 22 quantitative studies as measured by the medical education research study quality instrument (MERSQI).

**Quality assessment**	**Value**	**Publications references**
Mean MERSQI score	12.8	(51–69, 71–73)
MERSQI of the articles with a score of 13 and above	10	(51, 56, 57, 59, 60, 63, 66, 69, 71, 72)
Highest MERSQI score	17	(60)
Lowest MERSQI score	9.5	(67)

*NB, Scores on the MERSQI can range from 5 to 18, with a higher score indicating a higher-quality research study*.

Most curricula (20/24, 83.3%) used multiple educational modalities and 90% of studies turned out to be effective in achieving their primary outcomes (Table 4). A variety of teaching modalities were employed; didactics (53, 59, 61, 62, 64, 66, 70, 71, 73), small-group discussions (56–58, 61, 63, 66–68), reflection exercises (23, 56, 58, 66, 70) and simulations. (53, 57, 59, 62, 66, 68, 72) Likewise, virtual hangouts and technology-enhanced interventions (52, 58, 60, 69) were also significantly effective. A great majority of studies were conducted in multiple sessions within one academic year.

**Table 4:** Educational interventions with frequency and duration used in the selected studies (*n* = 24).

**Teaching modalities**	**No. (percentages) of studies**		**Publications references**	
	**Total no. (%)**	**Effective no. (%)^**a**^**	**^**a**^Effective**	**^**b**^Ineffective**
Single modality	4/24 (16.6%)	3/4 (75%)	(60, 69, 73)	(71)
Multimodality (Didactics, workshops, simulation, reflection)	20/24 (83.3%)	18/20 (90%)	(23, 51, 52, 54–59, 61, 63–68, 70, 72)	(53, 62)
Didactics (lectures, presentations, power-point, assessments, seminars, discussions)	9/24 (37.5%)	6/9 (66.6%)	(59, 61, 64, 66, 70, 73)	(53, 62, 71)
Small group/Case-Based discussion/workshops	8/24 (33.3%)	7/8 (87.5%)	(56–58, 61, 63, 66–68)	(62)
Simulation	7/24 (29.1%)	5/7 (71.4%)	(57, 59, 66, 68, 72)	(53, 62)
Role modeling/mentorship /interprofessional education	6/24 (25%)	6/6 (100%)	(23, 51, 52, 55, 65, 67)	
Reflective exercises (verbal or written)	5/24 (20.8%)	5/5 (100%)	(23, 56, 58, 66, 70)	
Technology enhanced learning (virtual patients, virtual hangouts, computerized tasks, hot spotting)	4/24 (16.6%)	4/4 (100%)	(52, 58, 60, 69)	
Meditation exercises	4/24 (16.6%)	3/4 (75%)	(54, 59, 64)	(71)
Early clinic exposure (student run clinic and experiential learning)	4/24 (16.6%)	4/4 (100%)	(23, 51, 55, 65)	
**Frequency and duration**	5/24 (20.8%)	4/5 (80%)	(59, 63, 70, 73)	(53)
One session (1–2 h)				
One half day or full day session	1/24 (2.4%)	1/1 (100%)	(60)	
Two-to-six sessions in an academic year	7/24 (29.1%)	7/7 (100%)	(52, 55, 56, 61, 64, 68, 72)	
More than six sessions in an academic year (e.g., a course, block rotation, longitudinal curriculum over a year)	7/24 (29.1%)	6/7 (85.7%)	(23, 54, 57, 58, 66, 67)	(71)
More than six sessions in multiple academic years (multiyear longitudinal curriculum)	4/24 (16.6%)	3/4 (75%)	(51, 65, 69)	(62)

a *Effective indicates that a statistically significant positive effect on the primary outcome was reported*.

b *Ineffective indicates that the reported effect was not statistically significant or statistical analysis was not reported*.

*NB, Individual studies used multiple types of educational interventions; therefore, number of studies are >24 and percentages add to >100*.

Only one study instituted the Balint training groups (57), while four studies (54, 59, 64, 71) used adapted mindfulness based curricular tools (75% effective ratio) developed by Kabat-Zinn (74). We did not observe any clear association between duration, frequency and complexity of an educational intervention and its effectiveness. For example, 75% (3/4) of single modality curricula were effective while multimodality curricula turned out to be effective in 90% (18/20) of the selected studies. Our data showed that single cross-sectional curricula (4/5) were as effective as longitudinal curricula (3/4) as shown in Table 4.

According to the four-level outcome-based Kirkpatrick model, 14/24 (58.3%) studies had a primary outcome pitching on level one; self-reported changes in attitudes and behavior (23, 53–56, 58, 61, 62, 64, 65, 67, 70, 71, 73). Two (4.8%) studies evaluated level three outcome (behavior), while six (25%) studies evaluated level four (patient outcomes) either by standardized or simulated patients or by a third party observation (51, 57, 60, 63, 66, 72). The leading constructs used in education interventions that showed positive impact on empathy and compassion of medical students in our study include communication skills, mindfulness, early clinical exposure, technology-enhanced learning, and humanities. A maximum of 10 studies used communication skills (53, 54, 56, 57, 60, 62, 63, 66, 71, 72), followed by mindfulness by 5 studies (54, 59, 64, 71), early clinical exposure by four (23, 51, 55, 65), technology-enhanced learning by virtual patient hangouts, computerized tasks, hot spotting by another four (52, 58, 60, 69), and comics (70), and arts and culture (61). Table 5 outlines an inventory of the validated instruments for outcome assessments used in the selected studies. The most commonly used self-assessment outcome tool was the Jefferson's Scale of Empathy by 15/24 (62.5%) studies (52–54, 56–58, 61–63, 65, 67, 69–72) that recruited 1,973 students. Conversely, 6/24 (25%) studies (54, 56, 59, 64, 69, 71) used a combination of tools to probe the impact of their interventions on behaviors and attitudes of medical students toward empathy and/or compassion and to determine the impact of self-compassion on behavior change. (59) Only three studies looked for the long-term effects of educational interventions (53, 62, 71).

**Table 5:** An inventory of the validated instruments for outcome assessments used in the selected studies (*n* = 24).

**Research instrument**	**Publications references**
**Empathy**	
Jefferson's scale of physician empathy	(52–54, 56–58, 61–63, 65, 67, 69–72)
Consultation and relational empathy measure	(57)
Toronto empathy questionnaire	(68)
Questionnaire of cognitive and affective empathy	(69)
**Attitude**	
Penn state college of medicine professionalism questionnaire	(56)
Life satisfaction questionnaire	(54)
Beliefs toward mental illness scale	(56)
Attitudes toward homelessness inventory	(52)
Basic personality traits inventory	(56)
Adapted altruism scale	(54)
**Students health and well-being**	
Brief differential emotions scale	(64)
Groningen reflection ability scale	(58)
Brief symptom inventory	(54)
Mental health continuum-short form	(54)
Five facet mindfulness questionnaire	(54)
Irrational beliefs inventory	(54)
Toronto mindfulness scale	(59, 64)
Marlowe-Crowne short form C	(64)
Self-compassion scale	(64)
Irrational beliefs inventory	(71)
Depression anxiety and stress scale	(54)
UCLA loneliness scale	(64)
Pittsburgh sleep scale	(64)
Substance use inventory	(64)
**Behavior**	
Micro expression training tool	(73)
Subtle expression training tool	(73)
Visual analog scale	(59)

## Discussion

Our study elucidates major findings of 24 studies that have collected data from 2,657 medical students using educational interventions on empathy and compassion. Overall, there is a positive impact of teaching empathy and compassion on medical students using a wide range of teaching pedagogies. The body of evidence from our research has deduced that effective communication skills, mindfulness, early clinical experience, comics, arts and culture, and technology-enhanced learning by virtual patients, hangouts and hot spotting had a positive impact on empathy and compassion of medical students.

The overarching findings in our study underpin a need to teach empathy using the concepts of physician-patient interactions, interprofessional practice with professional identity formation, stress awareness, and self-reflection and communication. In our study, a substantial body of evidence has stressed on empathetic communication for better patient outcomes (51, 57, 60, 63, 66, 72). Communication is the foremost determinant of a safe clinical practice and ensures satisfaction of both patients and healthcare providers (75). The outright benefits of timely and professional communication in the medical field are far ranging; greater patient understanding and compliance, superior clinical outcomes, improved patient safety and alleviation of patient stress (76). Kelley and Kelley have argued that verbal communication between a healthcare professional and a patient enacts an empathetic connection to the patient that carries a powerful transformative and healing power (77). Pity, sympathy, fellow-feeling, comfort, commiseration, assuagement, and reflexive encouragement constitute effective strands of practicing empathy and compassion (78). The identification of compassionate opportunities, conformation, and appreciation with pursuit provide a practice of compassionate and empathetic care (79). In the study by Beard et al. the researchers measured patients' satisfaction about clear communication by medical students comparing the Veterans Affairs Longitudinal Undergraduate (VALUE) program with a control group of patients matched with disease severity (51). The results of this longitudinal study on students' involvement using VALUE program (patient education, communication, and collaboration) and a control group without the VALUE program. The VALUE students showed significant improvement in care coordination among their patients and physicians. Educators should provide regular and sustainable opportunities to medical students to develop and validate their interpersonal communication skills that can potentially improve empathetic communication. The study by du Vaure et al. (57) used the Consultation And Relational Empathy Measure (CARE) scale in a two-site randomized controlled trial on medical students in a weekly Balint group forum for 2 months. Results of this group were compared with the group of routine education. There was an insignificant difference in mean CARE score (Intervention vs. control groups) however an increase in Jefferson Scale of Empathy (JSE) score for intervention and decrease in score for control from baseline to follow-up was recorded. LoSasso et al. have studied the impact of SALTED (Set-up, Ask, Listen, Type, Exceptions, Documentation) technique and role-plays in a case controlled trial on medical students using little educational time of 1 h only (63). At the start and end of clerkships, both groups completed the JSE. While faculty and standardized patients examined and graded students' history-taking and communication skills as well as their empathic behaviors using the Jefferson Scale of Patient Perceptions of Physician Empathy (JSPPPE) at the end of clerkships. The mean scores of JSE of both groups increased from pre-test to post-test. Historically, the Balint groups have been used to facilitate physicians and students in promoting and sustaining their empathy skills (12) This analysis reflects how the patient-related outcomes can be improved by using a wide range of educational tools and techniques (VALUE, CARE, and SALTED) for incorporating, measuring, and enhancing empathy and compassion in medical students.

In our research, a great majority of studies have measured self-reported changes in knowledge and skills of participants and only two studies could tap into behavioral changes after an intervention (59, 69). Sustained training sessions are essential for achieving such paradigm change in behaviors. Although literature has shown some controlled trials on empathy, a precursor for compassion, but there is scarce information on compassion training of medical students or healthcare professionals (80). One such compassion enhancing training exercise is provided by mindfulness, “a continuous awareness of present moment experience in a calm and non-judgmental manner” (81). Mindfulness is a multi-factorial construct that contains a host of correlates such as healthy lifestyle, health education, wellness, empathy, compassion, good quality of life, regular exercise, sleep and hygiene, yoga, and guided imagery (82). Training for enhancement of mindfulness can potentially enhance compassionate care in clinical practice (83), close physician-patient relationship and can help alleviate physician burnout (84). Mindful meditation, attention to breathing, didactic learning, reflection, mindful walking, journalism, and sitting meditation are some of the most popular mindfulness training exercises that have shown to enhance empathy and compassion in medical students and healthcare workers (59). In the study by van Dijik et al. (71), the authors incorporated a stress reduction training curriculum based on mindfulness strategies to improve the mental health of medical students during clinical clerkships. This intervention led to an insignificant betterment of mental health of students which was followed up longitudinally over a 20 month period. However, the researchers could not detect a difference in JSE at 12 months in the studied cohort of students. Such findings necessitate the incorporation of a sustained empathy training program, particularly empathetic communication, for undergraduate medical students.

Technology-enhanced learning has gained popularity in several branches of medical education. Likewise, virtual patients (VPs) and standardized patients (StPs) are used as attractive alternatives to real patients with promising results (58). Unfortunately, research has shown an empathy decline in medical students', particularly in the third year of medical school (85). At this stage, medical students enter clinical clerkships that witness their encounters with patients and their relatives. A suitable ramification to sustain empathy and compassion is the use of VPs and StPs that can provide a constant source for medical students' experiential learning and behavioral development without endangering patient safety. Experiential learning such as patient shadowing or wellness programs can potentially enhance cognitive and behavioral dimensions of empathy. The long-lasting impact of experiential learning is further endorsed by a study conducted by Modi et al. which showed that volunteering students had better empathy than the non-volunteered medial students (65), who showed a decline in empathy over time.

The intelligent use of arts, culture, and humanities in medical education is an emerging and promising approach that can revitalize the declining empathy in medical students (70). Comics “the combined use of images and text, sequentially, to tell a story, where the images complement and/or enhance the text” (86) is a powerful means of portraying complexities of medical knowledge. Graphical illustration with juxtaposed texts, depicts stories in a tangible manner which help to amalgamate the pictorial and textual cues highlighting an innovative use of technology enhanced learning and comics. Our research has shown some evidence that comics may serve as a distinct tool to promote empathy in medical education (87). Finally, interprofessional education and practice carries great potential to promote empathy and compassion by alleviating anxiety, stress and burnout associated with medical education (21, 88).

Our results highlight the fact that the educational interventions pitched at improving the knowledge outcomes can be significantly effective, followed by behaviors and finally, patient outcomes. This is understandable for some reasons; medical students are attuned to work hard to acquire knowledge which can be easily measured as an educational outcome. Behavioral change in students and improvement in patient outcomes demand a sustained interplay of generic and acquired traits and, therefore, it's hard to achieve. Another interesting finding of our research springs from the fact that frequency, duration and teaching modalities didn't have any bearing on the effectiveness of the educational intervention. Even single session interventions were as effective as longitudinal curricula and such curricula did not have a sustained and long-lasting impact. However, experiential learning drills lead to emotional and behavioral remodeling that can result in durable personality developments.

## Study Limitations

There are number of limitations of this review. The first relates to the comprehensiveness of the search and included articles. We searched four databases quite rigorously, but there remains a chance that certain pertinent studies are not captured by our search of databases, time and language restrictions. Second, though we aimed at highlighting the best practices in teaching empathy and/or compassion, this turned out to be difficult due to profound heterogeneity in the educational interventions and measurement tools used and the types of accomplished outcomes. Third, we planned to find a common curriculum for teaching and assessing empathy and compassion in medical education, its diverse and heterogeneous nature did not allow us to achieve our goal.

## Conclusion

In our systematic review, the identified 24 studies evaluated the empathy and/or compassion curricula for undergraduate medical students. There was a great diversity of teaching pedagogies, curriculum design, and duration of teaching that did not let us secure a single best-evidence teaching modality for empathy and/or compassion. Keeping the multidimensional construct nature in mind, a blend of teaching pedagogies is needed. However, major educational constructs of communication, mindfulness, self-care, self-regulation, reflective practice, early clinical exposure, technology-enhanced learning, comics and arts and culture should be targeted for teaching empathy and compassion. We found that even short standalone curriculum was as effective as longitudinal curriculum. In order to mitigate the risk of decline of empathy and compassion, a sustainable program rather than a single training activity is essential.

## Data Availability Statement

The original contributions presented in the study are included in the article/Supplementary Material, further inquiries can be directed to the corresponding author/s.

## Author Contributions

PM and SSG contributed substantially to conceiving the idea, created, reviewed, and validated the search strategy, hand searched and screened the titles and abstract, extracted, analyzed and interpreted the data and came up with the initial draft of manuscript. Later SYG individually evaluated the search strategy, tweaked the data mining process, revised and improved the intellectual content of the initial draft. PM, SYG, and SSG agreed to take responsibility for the final draft. All authors contributed to the article and approved the submitted version.

## Funding

This research was funded by the School of Postgraduate Studies and Research of the Royal College of Surgeons Ireland—Medical University Bahrain.

## Conflict of Interest

The authors declare that the research was conducted in the absence of any commercial or financial relationships that could be construed as a potential conflict of interest.

## Publisher's Note

All claims expressed in this article are solely those of the authors and do not necessarily represent those of their affiliated organizations, or those of the publisher, the editors and the reviewers. Any product that may be evaluated in this article, or claim that may be made by its manufacturer, is not guaranteed or endorsed by the publisher.
