# Supplementary material for: A Systematic Review of Educational Interventions and Their Impact on Empathy and Compassion of Undergraduate Medical Students
Source: Front Med (Lausanne). 2021 Nov 8;8:758377. doi: 10.3389/fmed.2021.758377 (PMC8606887; doi:10.3389/fmed.2021.758377)
Supplement: Supplementary file 1 [file Data_Sheet_1.docx]

**Appendix – Search Strategy**

**Search Strategy for Compassion – 22-07-2020**

**WEB OF SCIENCE**

| # | Searches | Results |
| --- | --- | --- |
| #1 | **TOPIC:**  (Compassion OR Empathy OR Caring)  *Indexes=SCI-EXPANDED, CPCI-S Timespan=All years* | 1,245,447 |
| #2 | **TOPIC:**  (Medical student)  *Indexes=SCI-EXPANDED, CPCI-S Timespan=All years* | 53,244 |
| #3 | **TOPIC:**  (Education OR Training OR Workshop OR Simulation)  *Indexes=SCI-EXPANDED, CPCI-S Timespan=All years* | 3,611,642 |
| #4 | **TOPIC:**  (Resident OR Physician)  *Indexes=SCI-EXPANDED, CPCI-S Timespan=All years* | 425,930 |
| #5 | **TOPIC:**  (Review OR Systematic review OR Scoping review OR Case report)  *Indexes=SCI-EXPANDED, CPCI-S Timespan=All years* | 2,874,569 |
| #6 | #3 AND #2 AND #1  *Indexes=SCI-EXPANDED, CPCI-S Timespan=All years* | 10,218 |
| #7 | #5 OR #4  *Indexes=SCI-EXPANDED, CPCI-S Timespan=All years* | 3,222,888 |
| #8 | #6 NOT #7  *Indexes=SCI-EXPANDED, CPCI-S Timespan=All years* | 4,575 |
| #9 | #6 NOT #7  **Refined by:** **PUBLICATION YEARS:** ( 2020 OR 2019 OR 2018 OR 2017 OR 2016 OR 2015 )  *Indexes=SCI-EXPANDED, CPCI-S Timespan=All years* | 2,012 |
| #10 | #6 NOT #7  **Refined by:** **PUBLICATION YEARS:** ( 2020 OR 2019 OR 2018 OR 2017 OR 2016 OR 2015 ) AND **DOCUMENT TYPES:** ( ARTICLE )  *Indexes=SCI-EXPANDED, CPCI-S Timespan=All years* | 1,809 |
| #11 | #6 NOT #7  **Refined by:** **PUBLICATION YEARS:** ( 2020 OR 2019 OR 2018 OR 2017 OR 2016 OR 2015 ) AND **DOCUMENT TYPES:** ( ARTICLE ) AND **LANGUAGES:** ( ENGLISH )  *Indexes=SCI-EXPANDED, CPCI-S Timespan=All years* | 1,739 |

**PubMed (n=762) 22-07-2020**

Search: **((((Compassion OR Empathy OR Caring AND (journalarticle[Filter])) AND (Medical student AND (journalarticle[Filter])))) AND (Education OR Training OR Workshop OR Simulation AND (journalarticle[Filter])) AND (journalarticle[Filter])) NOT ((Resident OR Physician AND (journalarticle[Filter])) OR (Review OR Systematic review OR Scoping review OR Case report AND (journalarticle[Filter])) AND (journalarticle[Filter]))** Filters: **Journal Article, from 2015 - 2020** Sort by: **Most Recent**

(((((((("empathy"[MeSH Terms] OR "empathy"[All Fields]) OR "compassion"[All Fields]) OR ("empathy"[MeSH Terms] OR "empathy"[All Fields])) OR (((((("care s"[All Fields] OR "cared"[All Fields]) OR "carefulness"[All Fields]) OR "cares"[All Fields]) OR "empathy"[MeSH Terms]) OR "empathy"[All Fields]) OR "caring"[All Fields])) AND "journal article"[Publication Type]) AND ((((("students, medical"[MeSH Terms] OR ("students"[All Fields] AND "medical"[All Fields])) OR "medical students"[All Fields]) OR ("medical"[All Fields] AND "student"[All Fields])) OR "medical student"[All Fields]) AND "journal article"[Publication Type])) AND (((((((((((((((((((((((("educability"[All Fields] OR "educable"[All Fields]) OR "educates"[All Fields]) OR "education"[MeSH Subheading]) OR "education"[All Fields]) OR "educational status"[MeSH Terms]) OR ("educational"[All Fields] AND "status"[All Fields])) OR "educational status"[All Fields]) OR "education"[MeSH Terms]) OR "education s"[All Fields]) OR "educational"[All Fields]) OR "educative"[All Fields]) OR "educator"[All Fields]) OR "educator s"[All Fields]) OR "educators"[All Fields]) OR "teaching"[MeSH Terms]) OR "teaching"[All Fields]) OR "educate"[All Fields]) OR "educated"[All Fields]) OR "educating"[All Fields]) OR "educations"[All Fields]) OR ((((((((("education"[MeSH Subheading] OR "education"[All Fields]) OR "training"[All Fields]) OR "education"[MeSH Terms]) OR "train"[All Fields]) OR "train s"[All Fields]) OR "trained"[All Fields]) OR "training s"[All Fields]) OR "trainings"[All Fields]) OR "trains"[All Fields])) OR (((("education"[MeSH Terms] OR "education"[All Fields]) OR "workshop"[All Fields]) OR "workshops"[All Fields]) OR "workshop s"[All Fields])) OR ((((((((((((((("computer simulation"[MeSH Terms] OR ("computer"[All Fields] AND "simulation"[All Fields])) OR "computer simulation"[All Fields]) OR "simulation"[All Fields]) OR "simul"[All Fields]) OR "simulate"[All Fields]) OR "simulated"[All Fields]) OR "simulates"[All Fields]) OR "simulating"[All Fields]) OR "simulation s"[All Fields]) OR "simulational"[All Fields]) OR "simulations"[All Fields]) OR "simulative"[All Fields]) OR "simulator"[All Fields]) OR "simulator s"[All Fields]) OR "simulators"[All Fields])) AND "journal article"[Publication Type])) AND "journal article"[Publication Type]) NOT ((((((((((((((((((("internship and residency"[MeSH Terms] OR ("internship"[All Fields] AND "residency"[All Fields])) OR "internship and residency"[All Fields]) OR "residencies"[All Fields]) OR "residency"[All Fields]) OR "reside"[All Fields]) OR "resided"[All Fields]) OR "residence"[All Fields]) OR "residence s"[All Fields]) OR "residences"[All Fields]) OR "residency s"[All Fields]) OR "resident"[All Fields]) OR "resident s"[All Fields]) OR "residents"[All Fields]) OR "resides"[All Fields]) OR "residing"[All Fields]) OR (((("physician s"[All Fields] OR "physicians"[MeSH Terms]) OR "physicians"[All Fields]) OR "physician"[All Fields]) OR "physicians s"[All Fields])) AND "journal article"[Publication Type]) OR (((((("review"[Publication Type] OR "review literature as topic"[MeSH Terms]) OR "review"[All Fields]) OR (("systematic review"[Publication Type] OR "systematic reviews as topic"[MeSH Terms]) OR "systematic review"[All Fields])) OR ((("scope"[All Fields] OR "scopes"[All Fields]) OR "scoping"[All Fields]) AND (("review"[Publication Type] OR "review literature as topic"[MeSH Terms]) OR "review"[All Fields]))) OR ("case reports"[Publication Type] OR "case report"[All Fields])) AND "journal article"[Publication Type])) AND "journal article"[Publication Type])

**Translations**

**Compassion:** "empathy"[MeSH Terms] OR "empathy"[All Fields] OR "compassion"[All Fields]

**Empathy:** "empathy"[MeSH Terms] OR "empathy"[All Fields]

**Caring:** "care's"[All Fields] OR "cared"[All Fields] OR "carefulness"[All Fields] OR "cares"[All Fields] OR "empathy"[MeSH Terms] OR "empathy"[All Fields] OR "caring"[All Fields]

**journalarticle[Filter]:** Journal Article[pt]

**Medical student:** "students, medical"[MeSH Terms] OR ("students"[All Fields] AND "medical"[All Fields]) OR "medical students"[All Fields] OR ("medical"[All Fields] AND "student"[All Fields]) OR "medical student"[All Fields]

**journalarticle[Filter]:** Journal Article[pt]

**Education:** "educability"[All Fields] OR "educable"[All Fields] OR "educates"[All Fields] OR "education"[Subheading] OR "education"[All Fields] OR "educational status"[MeSH Terms] OR ("educational"[All Fields] AND "status"[All Fields]) OR "educational status"[All Fields] OR "education"[MeSH Terms] OR "education's"[All Fields] OR "educational"[All Fields] OR "educative"[All Fields] OR "educator"[All Fields] OR "educator's"[All Fields] OR "educators"[All Fields] OR "teaching"[MeSH Terms] OR "teaching"[All Fields] OR "educate"[All Fields] OR "educated"[All Fields] OR "educating"[All Fields] OR "educations"[All Fields]

**Training:** "education"[Subheading] OR "education"[All Fields] OR "training"[All Fields] OR "education"[MeSH Terms] OR "train"[All Fields] OR "train's"[All Fields] OR "trained"[All Fields] OR "training's"[All Fields] OR "trainings"[All Fields] OR "trains"[All Fields]

**Workshop:** "education"[MeSH Terms] OR "education"[All Fields] OR "workshop"[All Fields] OR "workshops"[All Fields] OR "workshop's"[All Fields]

**Simulation:** "computer simulation"[MeSH Terms] OR ("computer"[All Fields] AND "simulation"[All Fields]) OR "computer simulation"[All Fields] OR "simulation"[All Fields] OR "simul"[All Fields] OR "simulate"[All Fields] OR "simulated"[All Fields] OR "simulates"[All Fields] OR "simulating"[All Fields] OR "simulation's"[All Fields] OR "simulational"[All Fields] OR "simulations"[All Fields] OR "simulative"[All Fields] OR "simulator"[All Fields] OR "simulator's"[All Fields] OR "simulators"[All Fields]

**journalarticle[Filter]:** Journal Article[pt]

**journalarticle[Filter]:** Journal Article[pt]

**Resident:** "internship and residency"[MeSH Terms] OR ("internship"[All Fields] AND "residency"[All Fields]) OR "internship and residency"[All Fields] OR "residencies"[All Fields] OR "residency"[All Fields] OR "reside"[All Fields] OR "resided"[All Fields] OR "residence"[All Fields] OR "residence's"[All Fields] OR "residences"[All Fields] OR "residency's"[All Fields] OR "resident"[All Fields] OR "resident's"[All Fields] OR "residents"[All Fields] OR "resides"[All Fields] OR "residing"[All Fields]

**Physician:** "physician's"[All Fields] OR "physicians"[MeSH Terms] OR "physicians"[All Fields] OR "physician"[All Fields] OR "physicians's"[All Fields]

**journalarticle[Filter]:** Journal Article[pt]

**Review:** "review"[Publication Type] .or. "review literature as topic"[MeSH Terms] .or. "review"[All Fields]

**Systematic review:** "systematic review"[Publication Type] .or. "systematic reviews as topic"[MeSH Terms] .or. "systematic review"[All Fields]

**Scoping:** "scope"[All Fields] OR "scopes"[All Fields] OR "scoping"[All Fields]

**review:** "review"[Publication Type] .or. "review literature as topic"[MeSH Terms] .or. "review"[All Fields]

**Case report:** "case reports"[Publication Type] .or. "case report"[All Fields]

**journalarticle[Filter]:** Journal Article[pt]

**journalarticle[Filter]:** Journal Article[pt]

**SCOPUS – (n=237) 22-07-2020**

( ( TITLE-ABS-KEY ( compassion  OR  empathy  OR  caring ) )  AND  ( TITLE-ABS-KEY ( medical  AND student ) )  AND  ( TITLE-ABS-KEY ( education  OR  training  OR  workshop  OR  simulation ) ) )  AND NOT  ( ( TITLE-ABS-KEY ( resident  OR  physician ) )  OR  ( TITLE-ABS-KEY ( review  OR  systematic  AND review  OR  scoping  AND review  OR  case  AND report ) ) )  AND  ( LIMIT-TO ( PUBYEAR ,  2020 )  OR  LIMIT-TO ( PUBYEAR ,  2019 )  OR  LIMIT-TO ( PUBYEAR ,  2018 )  OR  LIMIT-TO ( PUBYEAR ,  2017 )  OR  LIMIT-TO ( PUBYEAR ,  2016 )  OR  LIMIT-TO ( PUBYEAR ,  2015 ) )  AND  ( LIMIT-TO ( DOCTYPE ,  "ar" ) )  AND  ( LIMIT-TO ( SUBJAREA ,  "MEDI" )  OR  LIMIT-TO ( SUBJAREA ,  "HEAL" ) )  AND  ( LIMIT-TO ( LANGUAGE ,  "English" ) )

**EBSCO Host (n= 123) 22-07-2020**

( ( Compassion OR Empathy OR Caring ) AND Medical student AND ( Education OR Training OR Workshop OR Siulation ) ) NOT ( ( Resident OR Physician ) OR ( Review OR Systematic review OR Scoping review OR Case report ) )

**Limiters** - Publication Year: 2015-2020

**Expanders** - Apply equivalent subjects

**Narrow by Language:**- english

**Search modes** - Boolean/Phrase
